# Effects of selexipag and its active metabolite in contrasting the profibrotic myofibroblast activity in cultured scleroderma skin fibroblasts

**DOI:** 10.1186/s13075-018-1577-0

**Published:** 2018-05-02

**Authors:** Maurizio Cutolo, Barbara Ruaro, Paola Montagna, Renata Brizzolara, Emanuela Stratta, Amelia Chiara Trombetta, Stefano Scabini, Pier Paolo Tavilla, Aurora Parodi, Claudio Corallo, Nicola Giordano, Sabrina Paolino, Carmen Pizzorni, Alberto Sulli, Vanessa Smith, Stefano Soldano

**Affiliations:** 1 Research Laboratory and Academic Division of Clinical Rheumatology, Department of Internal Medicine, University of Genova, Polyclinic San Martino Hospital, Genoa, Italy; 2 Oncologic Surgery, Department of Surgery, Polyclinic San Martino Hospital, Genoa, Italy; 3 Department of Health Science, Unit of Dermatology, University of Genova, Polyclinic San Martino Hospital, Genoa, Italy; 4 Department of Medicine, Surgery and Neurosciences, Scleroderma Unit, University of Siena, Siena, Italy; 5 Department of Rheumatology, Ghent University Hospital, Ghent, Belgium

**Keywords:** Prostacyclin receptor agonists, Skin fibroblasts, Fibrosis, Systemic sclerosis, Connective tissue diseases

## Abstract

**Background:**

Myofibroblasts contribute to fibrosis through the overproduction of extracellular matrix (ECM) proteins, primarily type I collagen (COL-1) and fibronectin (FN), a process which is mediated in systemic sclerosis (SSc) by the activation of fibrogenic intracellular signaling transduction molecules, including extracellular signal-regulated kinases 1 and 2 (Erk1/2) and protein kinase B (Akt). Selexipag is a prostacyclin receptor agonist synthesized for the treatment of pulmonary arterial hypertension. The study investigated the possibility for selexipag and its active metabolite (ACT-333679) to downregulate the profibrotic activity in primary cultures of SSc fibroblasts/myofibroblasts and the fibrogenic signaling molecules involved.

**Methods:**

Fibroblasts from skin biopsies obtained with Ethics Committee (EC) approval from patients with SSc, after giving signed informed consent, were cultured until the 3^rd^ culture passage and then either maintained in normal growth medium (untreated cells) or independently treated with different concentrations of selexipag (from 30 μM to 0.3 μM) or ACT-333679 (from 10 μM to 0.1 μM) for 48 h. Protein and gene expressions of α-smooth muscle actin (α-SMA), fibroblast specific protein-1 (S100A4), COL-1, and FN were investigated by western blotting and quantitative real-time PCR. Erk1/2 and Akt phosphorylation was investigated in untreated and ACT-333679-treated cells by western botting.

**Results:**

Selexipag and ACT-333679 significantly reduced protein synthesis and gene expression of α-SMA, S100A4, and COL-1 in cultured SSc fibroblasts/myofibroblasts compared to untreated cells, whereas FN was significantly downregulated at the protein level. Interestingly, ACT-333679 significantly reduced the phosphorylation of Erk1/2 and Akt in cultured SSc fibroblasts/myofibroblasts.

**Conclusions:**

Selexipag and mainly its active metabolite ACT-333679 were found for the first time to potentially interfere with the profibrotic activity of cultured SSc fibroblasts/myofibroblasts at least in vitro, possibly through the downregulation of fibrogenic Erk1/2 and Akt signaling molecules.

## Background

Fibrosis is a common feature in diseases characterized by chronic tissue inflammation and damage, including systemic sclerosis (SSc) [1]. In SSc, fibrosis follows the microvascular/endothelial damage with activation of fibroblasts and their transition into profibrotic myofibroblasts [1–3]. After their phenotype transition, myofibroblasts acquire an increased persistent capability to synthesize and accumulate extracellular matrix (ECM) proteins, such as type I collagen (COL-1), and fibronectin (FN), beginning an aberrant “wound healing” process that contributes to fibrogenesis and finally to the systemic SSc fibrosis [4–6].

Myofibroblast transition and ECM overproduction are known to be induced by several profibrotic mediators, including transforming growth factor-β (TGFβ), endothelin-1 (ET-1), cytokines and chemokines (such as IL-6 and CCL18), whose circulating levels are increased in patients with SSc. These mediators have been shown to regulate the progression of fibrosis through the activation of intracellular signaling transduction pathways, such as those involving the activation of mitogen-activated protein kinase (MAPK), phosphatidylinositol 3-kinase (PI3K)/Akt and Wnt/β-catenin signaling [7–10]. Based on this knowledge, the blockage of fibroblast-to-myofibroblast transition and the attenuation of the profibrotic myofibroblast activity and related ECM overproduction might represent important steps in reducing the fibrotic process at least in SSc [11, 12].

Prostacyclin is an endothelium-derived eicosanoid that contributes to the maintenance of cardiovascular homeostasis, promotes both the proliferation and the differentiation of vascular smooth muscle cells, and acts as an inflammatory modulator [13]. The prostacyclin effects are mainly mediated by the activation of the prostacyclin I_2_ receptor (IP receptor) and the subsequent accumulation of intracellular cyclic adenosine monophosphate (cAMP) [14]. The IP receptor is a member of the G protein coupled receptor superfamily highly expressed in several cell types, including vascular smooth muscle cells, leukocytes, monocytes, and fibroblasts [14–18].

Selexipag is an IP receptor agonist characterized by a non-prostanoid structure and it has been synthesized for the treatment of pulmonary arterial hypertension (PAH) [19]. Selexipag has one major active metabolite, ACT-333679, which is also a selective IP receptor agonist, and both compounds are characterized by specific binding and high affinity to this receptor [20].

This study investigated the effects of selexipag and ACT-333679 in reducing the activity of cultured skin SSc fibroblasts and their ECM protein overproduction, through the ability to interfere with the activation of those intracellular signaling transduction molecules involved in the regulation and progression of fibrosis, primarily MAPK family members (i.e. extracellular signal-regulated kinases 1 and 2 (Erk1/2)) and protein kinase B (PKB or Akt).

## Methods

### Patients with SSc and HSs

Six female patients with SSc (mean age 63 ± 10 years), who fulfilled the new European League Against Rheumatism (EULAR)/American College of Rheumatology (ACR) criteria for SSc, and five sex-matched healthy volunteer subjects (HSs) (mean age 57 ± 8 years) were recruited from the Divisions of Rheumatology and Dermatology at the University of Genova in accordance with the Declaration of Helsinki, after providing signed informed consent and following the local Ethical Board Committee approval (protocol ID: 237REG2015) [21]. Patients with SSc had an “active” nailfold videocapillaroscopic (NVC) pattern and their demographic and clinical characteristics are summarized in Table 1.

**Table 1:** Demographic and clinical characteristics of patients with systemic sclerosis (SSc)

	Sample ID					
Parameters	SSc1	SSc2	SSc3	SSc4	SSc5	SSc6
Age	51	58	56	78	65	72
Sex	F	F	F	F	F	F
RP duration (months)	108	168	36	720	180	288
Disease duration (months)	108	168	24	48	36	288
Skin involvement	lcSSc	lcSSc	lcSSc	lcSSc	lcSSc	lcSSc
mRSS score	17	4	14	14	5	17
VCP pattern	Active	Active	Active	Active	Active	Active
ANA	Positive	Positive	Positive	Positive	Positive	Positive
Autoantibody	Scl70	CENP	Fibrillarin	CENP	CENP	CENP
Organ involvement	Yes (lung, esophagus)	No	No	No	No	No
Therapy	Ca_2+_ antagonists, ACE inhibitors, immunosuppressors, ERAs, aspirin, vitamin D	Aspirin, aminaphtone, angiotensin receptor blockers	Aminaphtone, vitamin D	Aspirin, steroids, ACE inhibitors, aminaphtone, MTX	Aspirin	Aminaphtone, vitamin D

Demographic and clinical parameters of patients with SSc enrolled into the study

*F* female, *RP* Raynaud’s phenomenon, *lcSSc*  “limited” cutaneous systemic sclerosis skin involvement, *mRSS* modified Rodnan skin score, *VCP* nailfold videocapillaroscopy, *ANA* anti-nuclear antibody, *Scl70* anti-topoisomerase antibody, *CENP* anti-centromere antibody, *ACE* angiotensin converting enzyme, *ERA* endothelin receptor antagonist, *MTX* methotrexate

### Cell cultures and treatments

Skin fibroblasts were isolated from full­thickness biopsies of the clinically involved skin of one third of the distal forearm of patients with SSc and of HSs, in accordance with the EULAR scleroderma trials and research (EUSTAR) protocol and our recent studies [5, 22, 23].

For the in vitro experiments, human SSc and HS skin fibroblasts were grown in Roswell Park Memorial Institute (RPMI) 1640 medium supplemented with 10% fetal bovine serum (FBS), 1% penicillin-streptomycin, and L-glutamine (Lonza Clonetic, Switzerland) and used at the 3^rd^ culture passage.

The fibroblasts isolated from the biopsy sample from each patient with SSc were independently cultured up to 80% of confluence and then maintained in serum-free medium for 4 h. After starvation, a portion of these cells was maintained in growth medium at 5% of FBS without treatment (untreated cells) and another portion was treated for 48 h with three different concentrations of selexipag (30 μM, 3 μM, or 0.3 μM); a further portion of cells was treated for 48 h with three different concentrations of ACT-333679 (10 μM, 1 μM or 0.1 μM) (Actelion Pharmaceutics, Switzerland), in accordance with recent studies [15, 24–26].

The fibroblasts isolated from the biopsy sample from each HS were cultured up to 80% of confluence and maintained in growth medium at 5% FBS for 48 h, after starvation in serum-free medium for 4 h.

To assess the activation of intracellular signaling transduction molecules involved in the regulation of fibrosis, human SSc skin fibroblasts were cultured up to 80% of confluence and after starvation in serum-free medium for 4 h, the cells were maintained in growth medium at 5% FBS without any treatment (untreated cells) or treated with three different concentrations of ACT-333679 (10 μM, 1 μM, or 0.1 μM, Actelion Pharmaceutics) for 15 min, 30 min, and 48 h.

### Immunocytochemistry analysis

SSc and HS skin fibroblasts were cultured in Flexi PERM chamber slides (5 × 10^3^ cells/spot) (Millipore, Billerica, MA, USA) and treated as described in “Cell cultures and treatments”. At the end of treatment, the cells were fixed in 2% paraformaldehyde and incubated with primary antibodies (dilution 1:100) to human α-smooth muscle actin (α-SMA) (Dako Cytomation, Denmark), COL-1 (Enzo Life Science, UK), and FN (Sigma-Aldrich, Milan, Italy). Linked antibodies were detected by biotinylated universal secondary antibody and then with the HRP-streptavidine complex (Vector Laboratories, CA, USA). The analysis of α-SMA expression and ECM protein synthesis was performed in each experimental condition evaluating the same number of cells by light microscopy (magnification × 20) (Leica, Cambridge, UK).

### Western blotting

For the evaluation of myofibroblast phenotype markers and ECM proteins, cultured human SSc skin fibroblasts were lysed with NucleoSpin RNA/protein (Macherey­Nagel, Duren, Germany), whereas to assess the Erk1/2 and Akt activation, cultured cells were lysed in ice using Radio-Immunoprecipitation Assay (RIPA) buffer (HEPES 20 mM, NaCl 0.15 M, Glycerol 10%, EDTA 1 mM, aprotinin 10 μg/ml, leupeptin 10 μg/ml, pepstatin 1 μg/ml, PMSF 1 mM, Na_3_Vo_4_ 1 mM, Sigma-Aldrich). The protein quantification was performed by the Bradford method. For every condition, 15 μg of protein was separated by electrophoresis on 8% and 4–16% gradient tris­glycine gels (GenScript, New York, NY, USA) and transferred onto Hybond-C-nitrocellulose membrane (Life Technologies Ltd., Paisley, UK). After 1 h in a phosphate buffer solution (PBS)1x containing triton­X 0.1% and non­fat powdered milk 5% (Sigma-Aldrich), the membranes were incubated overnight at 4 °C with primary antibodies anti-human α-SMA (dilution 1:1000; Cell Signaling, MA, USA), fibroblast specific protein-1 (FSP1 or S100A4, dilution 1:500; Santa Cruz Biotechnology, CA, USA), COL-1 (dilution 1:600; Enzo Life Science), and FN (dilution 1:1000; Sigma-Aldrich).

Primary antibodies anti-human phospho-Erk1/2 (dilution 1:2000), Erk1/2 (dilution 1:600), phospho-Akt (dilution 1:300), and Akt (dilution 1:500) (Santa Cruz Biotechnology) were used to investigate the activation of intracellular signaling transduction pathways. The membranes were subsequently incubated with secondary antibodies (dilution 1:2000; Cell Signaling) and also incubated with primary HRP-conjugated antibody to human glyceraldehyde 3-phosphate dehydrogenase (GAPDH) (dilution 1:5000; Cell Signaling) to confirm similar loading of protein samples into the gels and the efficiency in the electrophoretic transfer.

Protein synthesis was detected using the enhanced chemiluminescence system (Luminata Crescendo, Millipore) and the densitometric analysis was performed by the UVITEC Image Analysis System (UVITEC, Cambridge, UK). Western blotting was performed in six independent experiments on cultured human SSc skin fibroblasts.

### Quantitative real-time polymerase chain reaction (qRT-PCR)

Total RNA was obtained from cultured human SSc skin fibroblasts using NucleoSpin RNA/protein (Macherey-Nagel) and quantified by nanodrop, which was also used to evaluate the RNA integrity, in accordance with the manufacturer’s protocol (Thermo Scientific, Wilmington, USA). For each experimental condition, first­strand complementary DNA (cDNA) was synthesized from 1 μg of total RNA using QuantiTect Reverse Transcription Kit (Qiagen, Milan, Italy).

The qRT-PCR was performed on an Eppendorf Realplex 4 Mastercycler using Real MasterMix SYBR Green detection system (Eppendorf, Milan, Italy) in a total volume of 10 μL loaded in triplicate. Primers for *α-SMA* (NM_001613), *S100A4* (NM_002961), *COL-1* (NM_000088), *FN* (NM_002026), and *β­actin* (NM_001101, housekeeping gene) were supplied by Primerdesign (Primerdesign, UK).

The gene expression values were calculated using the comparative ΔΔ cycle threshold (ΔΔCT) method and corresponded to the expression level (fold increase) of the target gene compared to untreated cells, taken as the unit value [27]. In all qRT-PCR assays, the melting curve was performed to confirm the specificity of the SYBR green assay, and the qRT-PCR was performed on six independent experiments on cultured human SSc skin fibroblasts.

### Statistical analysis

The statistical analysis was carried out by the non-parametric Mann-Whitney test to compare unpaired treatments. Any *p* value lower than 0.05 was considered as statistically significant. Results of western blotting and qRT-PCR were analyzed as mean ± standard deviation (SD).

## Results

### Selexipag and ACT-333679 reduced α-SMA and S100A4 protein synthesis and gene expression in cultured human SSc skin fibroblasts

**Fig. 1:**
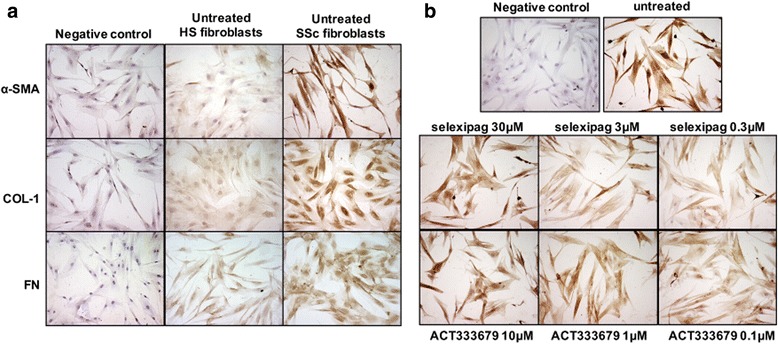
Evaluation of α-smooth muscle actin (α-SMA), type I collagen (COL-1), and fibronectin (FN) in cultured human skin fibroblasts isolated from patients with systemic sclerosis (SSc) and healthy subjects (HSs). **a** Immunocytochemistry analysis of α-SMA, COL-1 and FN in cultured human SSc and HS skin fibroblasts maintained in normal growth medium without treatment for 48 h. **b** Immunocytochemistry analysis of α-SMA in cultured human SSc skin fibroblasts maintained in normal growth medium (untreated), treated with selexipag at the concentration of 30 μM, 3 μM, and 0.3 μM, and treated with ACT-333679 at the concentration of 10 μM, 1 μM, and 0.1 μM for 48 h

Cultured SSc skin fibroblasts maintained in growth medium for 48 h without treatment (untreated cells) had increased expression of α-SMA compared to cultured HS fibroblasts, indicating their ongoing differentiation and transition into activated myofibroblasts (Fig. 1a). In cultured SSc fibroblasts, selexipag and ACT-333679 reduced the protein expression of α-SMA compared to untreated cells at all tested concentrations, as observed by immunocytochemistry analysis (Fig. 1b).

In these cells, the effects of selexipag and ACT-333679 on both the protein synthesis and the gene expression of α-SMA and S100A4 (specific myofibroblast markers) were evaluated by western blotting and qRT-PCR.

**Fig. 2:**
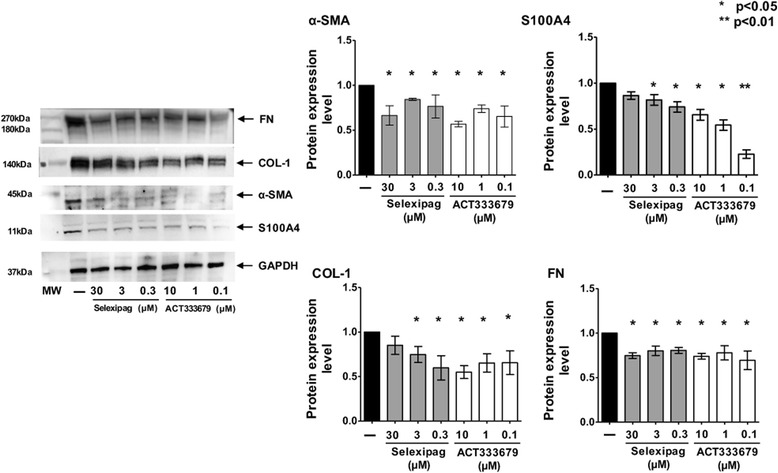
Evaluation of the protein synthesis of myofibroblast phenotype markers and extracellular matrix macromolecules in cultured human systemic sclerosis (SSc) skin fibroblasts. Western blotting and related densitometric analysis of the protein synthesis of α-smooth muscle actin (α-SMA), S100A4, type I collagen (COL-1) and fibronectin (FN) in cultured human SSc skin fibroblasts maintained in normal growth medium (untreated), treated with selexipag at the concentration of 30 μM, 3 μM, and 0.3 μM, and treated with ACT-333679 at the concentration of 10 μM, 1 μM, and 0.1 μM, for 48 h. For each experimental condition, the value of the synthesis of α-SMA, S100A4, COL-1, and FN was normalized to that of the corresponding glyceraldehyde 3-phosphate dehydrogenase (GAPDH). The resulting value with each treatment was compared to that of the untreated cells (taken as a unit value), in order to obtain the level of protein synthesis. A molecular weight (MW) lane was also included. The final results represent the mean ± standard deviation (SD) of the values obtained from six independent experiments on cultured human SSc skin fibroblasts: *p < 0.05 and **p < 0.01 vs. untreated cells. kDa, kiloDalton

Selexipag significantly reduced the protein synthesis of α-SMA at all tested concentrations compared to untreated cells (*p* < 0.05 for all) and that of S100A4 at the concentration of 3 μM and 0.3 μM (*p* < 0.05 compared to untreated cells) (Fig. 2).

Similarly to selexipag, all tested concentrations of ACT-333679 significantly reduced the protein synthesis of α-SMA and S100A4 compared to untreated cells (α-SMA, *p* < 0.05 for all concentrations; S100A4, *p* < 0.05 for ACT-333679 10 μM and 1 μM, *p* < 0.01 for ACT-333679 0.1 μM) (Fig. 2).

**Fig. 3:**
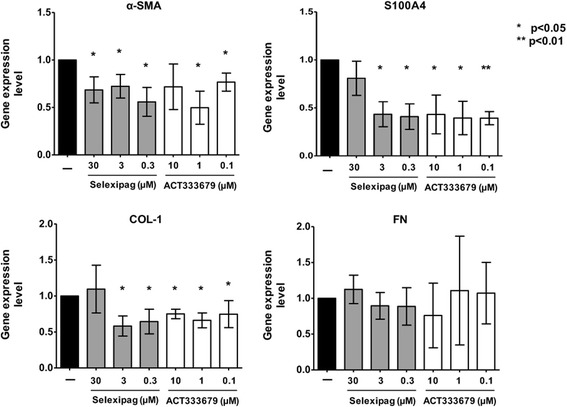
Evaluation of the gene expression of myofibroblast phenotype markers and extracellular matrix macromolecules in cultured human systemic sclerosis (SSc) skin fibroblasts. Quantitative real-time polymerase chain reaction (qRT-PCR) and related analysis of the gene expression of α-smooth muscle actin (*α-SMA*), *S100A4*, type I collagen (*COL-1*) and fibronectin (*FN*) in cultured human SSc skin fibroblasts maintained in normal growth medium (untreated), treated with selexipag at the concentration of 30 μM, 3 μM, and 0.3 μM, and treated with ACT-333679 at the concentration of 10 μM, 1 μM, and 0.1 μM, for 48 h. The final results represent the mean ± standard deviation (SD) of the values obtained from six independent experiments on cultured human SSc skin fibroblasts: *p < 0.05 and **p < 0.01 vs. untreated cells

At the gene expression level, selexipag 30 μM, 3 μM, and 0.3 μM significantly downregulated *α-SMA* (*p* < 0.05 for all), whereas only the concentrations of 3 μM and 0.3 μM induced significant downregulation in the gene expression of *S100A4* compared to untreated cells (*p* < 0.05) (Fig. 3).

At the same time, ACT-333679 downregulated the gene expression of these myofibroblast phenotype markers. ACT-333679 1 μM and 0.1 μM significantly reduced the gene expression of *α-SMA* compared to untreated cells (*p* < 0.05, for both concentrations), whereas the downregulation induced by ACT-333679 10 μM was not statistically significant (Fig. 3). However, all three tested concentrations of ACT-333679 significantly downregulated the gene expression of *S100A4* compared to untreated cells (*p* < 0.05 for ACT-333679 10 μM and 1 μM; *p* < 0.01 for ACT-333679 0.1 μM) (Fig. 3).

### Selexipag and ACT-333679 decreased the ECM protein synthesis and gene expression in cultured human SSc skin fibroblasts

As observed on immunocytochemistry analysis, cultured SSc skin fibroblasts maintained in growth medium for 48 h without treatment (untreated cells) had increased production of both COL-1 and FN compared to HS skin fibroblasts, confirming their ongoing activated profibrotic phenotype (Fig. 1a).

In cultured SSc fibroblasts, selexipag and ACT-333679 reduced the synthesis of both COL-1 and FN compared to untreated cells (Fig. 2). Selexipag 3 μM and 0.3 μM and ACT-333679 10 μM, 1 μM, and 0.1 μM induced significant reduction of COL-1 synthesis (*p* < 0.05 vs. untreated cells), whereas FN production was significantly decreased by all tested concentrations of selexipag and ACT-333679 (*p* < 0.05 vs. untreated cells, for all concentrations of both compounds) (Fig. 2). All these data were obtained by western blotting.

At the gene expression level, selexipag 3 μM and 0.3 μM significantly downregulated *COL-1* compared to untreated cells (*p* < 0.05 for both) (Fig. 3). ACT-333679 10 μM, 1 μM, and 0.1 μM significantly downregulated the gene expression of *COL-1* compared to untreated cells (*p* < 0.05 for all) (Fig. 3). No significant modulatory effects of selexipag and ACT-333679 were observed on *FN* gene expression (Fig. 3).

### ACT-333679 reduced both Erk1/2 and Akt phosphorylation in cultured human SSc skin fibroblasts

Since ACT-333679 is the major active metabolite of selexipag, its possible modulatory effect on Erk1/2 and Akt activation was directly investigated in cultured SSc skin fibroblasts.

**Fig. 4:**
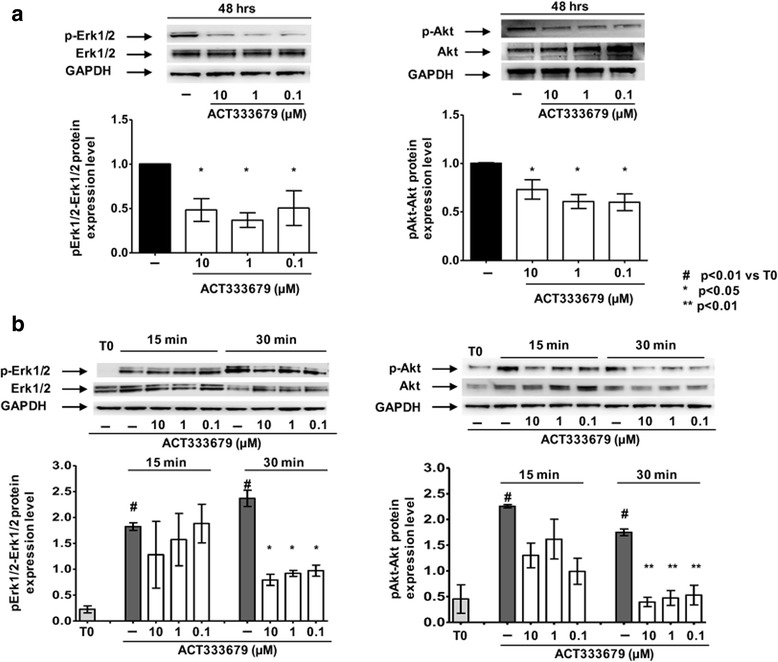
Evaluation of the extracellular signal-regulated kinases 1 and 2 (Erk1/2) and protein kinase B (Akt) activation in cultured human systemic sclerosis (SSc) skin fibroblasts. **a** Western blotting and related densitometric analysis of phospho-Erk1/2 (p-Erk1/2), Erk1/2, phospho-Akt (p-Akt) and Akt in cultured human SSc skin fibroblasts maintained in normal growth medium (untreated) or treated with ACT-333679 at the concentration of 10 μM, 1 μM, and 0.1 μM for 48 h. The expression of phosphorylated proteins (p-Erk1/2 and p-Akt) was first normalized to that of the naïve proteins (Erk1/2 and Akt) and then normalized to that of the related expression of glyceraldehyde 3-phosphate dehydrogenase (GAPDH). The resulting value of each treatment was compared to that of the untreated cells (taken as the unit value): *p < 0.05 vs. untreated cells. **b** Western blotting and related densitometric analysis of phospho-Erk1/2 (p-Erk1/2), Erk1/2, phospho-Akt (p-Akt) and Akt, in cultured human SSc skin fibroblasts maintained in normal growth medium (untreated) or treated with ACT-333679 at the concentration of 10 μM, 1 μM, and 0.1 μM for 15 min and 30 min. The expression of phosphorylated proteins (p-Erk1/2 and p-Akt) was normalized to that of the naïve proteins (Erk1/2 and Akt). The resulting value was then normalized to that of the related expression of GAPDH: ^#^*p* < 0.01 vs. time zero (T0); *p < 0.05 and **p < 0.01 vs. untreated cells. The final results represent the mean ± standard deviation (SD) of the values obtained from six independent experiments on cultured human SSc skin fibroblasts

Cultured untreated SSc fibroblasts showed the activation of both Erk1/2 and Akt at 48 h, as observed through their increased phosphorylation state (Fig. 4a). Interestingly, ACT-333679 significantly reduced the phosphorylation of both Erk1/2 and Akt compared to untreated cells (*p* < 0.05 for all concentrations) (Fig. 4a). Moreover, cultured SSc fibroblasts were treated for 15 min and 30 min in the presence or absence of ACT-333679, in order to understand whether these mediators were activated early.

It was interesting to note that cultured untreated SSc fibroblasts showed a significant increase in Erk1/2 phosphorylation after 15 min and 30 min (*p* < 0.01 vs. time zero (T0) for all time points), whereas ACT-333679 treatment was shown to significantly reduce Erk1/2 phosphorylation after 30 min of treatment (*p* < 0.05 vs. untreated cells for all tested concentrations) (Fig. 4b).

At the same time, cultured untreated SSc fibroblasts had a rapid and significant increase in Akt phosphorylation after 15 min and 30 min (*p* < 0.01 vs. T0 for both time points), and ACT-333679 already contrasted this increased Akt phosphorylation after 15 min, and significantly after 30 min of treatment (*p* < 0.01 vs. untreated cells, for all tested concentrations) (Fig. 4b).

## Discussion

The results of the study show for the first time that selexipag and mainly its active metabolite ACT-333679 can potentially downregulate the profibrotic activity of cultured SSc fibroblasts/myofibroblasts through the reduction of Erk1/2 and Akt phosphorylation/activation at least in vitro.

The effect was characterized after treatment by the reduced synthesis of specific myofibroblast markers, such as α-SMA and S100A4, and then of ECM proteins, COL-1 and FN.

Of note, cultured SSc skin fibroblasts, maintained for 48 h in growth medium, expressed high levels of α-SMA compared to cultured HS skin fibroblasts, confirming their ongoing transition into activated myofibroblasts.

Therefore, the ability of selexipag and ACT-333679 to downregulate the gene expression and the protein synthesis of α-SMA and S100A4, seems to suggest a possible role for this IP receptor agonist to interfere with the fibroblast-to-myofibroblast transition.

It is recognized that in wound healing and in fibrotic diseases, including SSc, the transition of fibroblasts into profibrotic α-SMA^+^myofibroblasts is a fundamental event, making these cells the key mediators of fibrogenesis [28, 29]. The profibrotic capability of myofibroblasts is linked to their overproduction and deposition of ECM macromolecules, primarily COL-1 and FN, in the affected tissues [30, 31]. The present in vitro study confirmed that cultured SSc α-SMA^+^fibroblasts are characterized by greater ECM protein synthesis compared to cultured HS fibroblasts.

Therefore, the ability of selexipag and ACT-333679 to significantly reduce the protein synthesis of COL-1 and FN in cultured SSc skin fibroblasts/myofibroblasts might suggest a possible antifibrotic action of the IP receptor agonist.

Selexipag and ACT-333679 bind selectively and with higher affinity to the IP receptor compared to other prostacyclin analogs (such as beraprost or treprostinil), targeting the pathway of prostacyclin activation and leading to vasodilatory and anti-proliferative effects, as observed in several mouse models of PAH [20, 30, 31]. In addition, ACT-333679 is present at 3-fold to 4-fold higher levels and is approximately 37-fold more potent in inducing in vitro activation of human IP receptor than the parent compound, suggesting that the metabolite is the major one responsible for the selexipag effects in humans [32, 33]. In accordance with these observations, the results of the present in vitro study showed that in cultured SSc skin fibroblasts/myofibroblasts, ACT-333679 induced the same effects as selexipag, but at a concentration three times lower than that of the parent compound (selexipag 3 μM and 0.3 μM, ACT-333679 1 μM and 0.1 μM). The concentrations of selexipag and especially ACT-333679 were in accordance with several in vitro studies [15, 24–26]. Moreover, the lowest ACT-333679 concentration (0.1 μM) is in range with the values of its bioavailability which were well-tolerated by the HSs and were indicated by the area under the curve during a dose interval of administration (AUC_t_), as reported in several studies [25, 26].

It is interesting to note that selexipag and ACT-333679 did not have a dose-dependent effect in reducing the profibrotic activity of cultured SSc skin fibroblasts/myofibroblasts. A possible explanation of this might be related to the intrinsic variability in the cellular response to treatments with selexipag and ACT-333679 in cultured skin fibroblasts isolated from each enrolled patient with SSc. Therefore, the variability might be reduced by increasing the number of patients with SSc and the related experiments.

Another possible explanation for the absence of a dose-response effect might depend on the expression level and activity of the IP receptor on the cell surface of cultured SSc skin fibroblasts/myofibroblasts. In a recent study it has been observed that several cell types, including pulmonary arterial smooth muscle cells (PASMCs), displayed a moderate degree of constitutive IP receptor internalization and treatment with prostacyclin analogs (such as beraprost, treprostinil) induced strong depletion of this receptor from the cell surface, whereas the non-prostanoid agonists selexipag and ACT-333679 did not induce these effects [15]. Moreover, ACT-333679 1 μM and 10 μM induced the same increase in the expression of the IP receptor on the cell surface, which was higher than that induced by ACT-333679 0.1 μM [15]. Based on this observation, it might be possible to hypothesize that the concentrations of selexipag 0.3 μM and 3 μM and ACT-333679 0.1 μM and 1 μM might be sufficient to bind to all the IP receptors on the cell surface, inducing the activation of an intracellular signaling cascade that involves increase in cAMP production. On the other hand, ACT-333679 at the concentration of 0.1 μM, 1 μM, and 10 μM induced overlapping effects in terms of cAMP production and efficacy to contrast proliferation and fibrosis-related readouts in human PASMCs in a saturable manner, determining an efficacy plateau [15].

Another important result of our in vitro study is that selexipag and ACT-333679 exerted their effects efficiently targeting the profibrotic activity of fibroblasts/myofibroblasts of the skin, which represents one of the tissues affected by the fibrotic process. These observations suggest that prostacyclin pathways may play an important role in fibrotic diseases, including pulmonary fibrosis, as already observed for the prostacyclin analog iloprost, which reduces COL-1 synthesis through the increase in cAMP production in SSc skin fibroblasts [34].

FN is a multifunctional macromolecule produced by fibroblasts that can modulate fibroblast-mediated collagen gel contraction. According to this process, the activation of the IP receptor was recently shown to inhibit FN release and fibroblast-mediated collagen gel contraction, suggesting its possible contribution in tissue remodeling through the action on fibroblasts [35]. The effects of selexipag and ACT-333679, as observed at 48 h and limited to the reduction in protein synthesis, might suggest that their downregulatory effects on FN gene expression should be a very early event, as already observed in the in vitro study by Kamio et al. [35]. In this study the authors demonstrated how the prostacyclin analog beraprost induced downregulation of FN messenger RNA (mRNA) expression after 5 h of treatment, whereas the reduction in FN release was observed after 48 h of treatment [35]. This result might be due to the activation of post-transcriptional mechanisms induced by prostacyclin analogs and IP receptor agonists, which may be interesting to investigate. Based on this observation, even shorter in vitro experiments should be performed (at 4 h and 12 h).

Several molecules and growth factors contribute to the induction of the profibrotic phenotype of fibroblasts, including TGFβ1, ET-1 and cytokines (such as IL-6), through the activation of fibrogenic intracellular signaling pathways [7]. Among the molecules involved in these intracellular signaling pathways, Erk1/2 and Akt play an important role in mediating the effects of TGFβ1 and ET-1, which are considered key inducers of the fibrotic process and the levels of which are increased in SSc [7, 36]. As recently demonstrated, cultured SSc fibroblasts are characterized by increased phosphorylation of Erk1/2 and Akt when compared to HS fibroblasts suggesting the involvement of these molecules in mediating the activated profibrotic phenotype of SSc myofibroblasts [36].

The results of our in vitro study showed that cultured SSc fibroblasts/myofibroblasts are characterized by early phosphorylation and related activation of both Erk1/2 and Akt (15 min and 30 min) that was still present at a later time point (48 h).

Erk1/2 are members of the MAPK family implicated in several profibrotic signaling pathways [7, 37]. Previous investigations suggested that the activation of Erk1/2, induced by TGFβ1, is responsible for the overexpression of α-SMA and COL-1 in cultured fibroblasts, promoting the transition of these cells into profibrotic myofibroblasts [38]. In addition, several studies have reported that Erk1/2 can mediate the ET-1-induced expression of collagen isoforms and contractile proteins involved in the myofibroblast contraction and migration through the activation of the activator protein-1 (AP-1) transcription factor, contributing to the profibrotic effects of ET-1 [39–41].

Akt is a serine-threonine kinase that can engage multiple downstream signaling substrates and pathways, including the PI3K pathway, a Smad-independent intracellular signaling pathway that mediates TGFβ-induced fibrosis [7]. Very recent studies showed that the phosphorylation and the related activation of Akt are primarily implicated in fibroblast/myofibroblast differentiation, migration and proliferation of pulmonary and cardiac fibroblasts stimulated with TGFβ and ET-1 [42, 43].

Moreover, in previous investigations, the inhibition of PI3K/Akt phosphorylation was found to interfere with the expression of α-SMA in SSc lung fibroblasts and the ability of these cells to contract the collagen gel matrix mediated by ET-1 [44, 45].

Accordingly, the ability of ACT-333679 to significantly reduce the phosphorylation of both Erk1/2 and Akt in cultured SSc fibroblasts/myofibroblasts found in our study, seems to further suggest that the effects of this IP receptor agonist on these cells might involve downregulation in the profibrotic signaling pathways.

One limitation of the study is that the antifibrotic effects of selexipag and ACT-333679 were investigated in in vitro cultures of human skin fibroblasts, so they should also be replicated on lung fibroblasts, since this IP receptor agonist was synthesized mainly for the treatment of PAH.

If confirmed on lung fibroblasts, the vasodilator activity exerted by selexipag in SSc patients with PAH should be integrated by important antifibrotic lung effects, possibly exerting disease-modifying effects over the long term. Moreover, to give an overview of selexipag and its vasodilator effect, it is necessary to cite a recent randomized, placebo-controlled phase II study that showed how selexipag did not reduce the number of Raynaud’s phenomenon (RP) attacks compared to placebo in adults with RP secondary to SSc, probably due to the differences between vascular effects in the systemic circulation compared to the pulmonary vasculature [46]. It is interesting to note that other therapies targeting the prostacyclin pathways can have positive or negative effects on RP based on the route of administration; in accordance with this observation, different studies highlighted that intravenous iloprost administration showed efficacy in reducing the number, severity, and duration of RP attacks whereas oral administration did not have any of these effects [47, 48]. Therefore, the route of administration of selexipag and other molecules targeting prostacyclin pathway may have an impact on the efficacy as indicated by the treatment response.

In addition, since Erk1/2 and Akt have been demonstrated to contribute to the intracellular signaling pathways responsible for the profibrotic effects exerted by TGFβ1 and ET-1, it might be interesting to investigate whether selexipag and ACT-333679 may contrast this action in cultured fibroblasts, both alone and in combination with selective TGFβ1 signaling blocking agents or ET-1 receptor antagonists, such as macitentan.

Moreover, based on present results, a new study is currently in progress to investigate the functional relevance of selexipag and its active metabolite, evaluating their ability to reduce the enhanced contractile fibrotic phenotype of SSc skin fibroblasts/myofibroblasts.

## Conclusions

In conclusion, the in vitro study showed, for the first time, the potential antifibrotic effects of selexipag and its active metabolite in cultured SSc skin fibroblasts/myofibroblasts that might be determined through interference in the activation of the Erk1/2 and Akt signaling pathways. The action was characterized by the reduction in specific markers of activated myofibroblast phenotype and the ECM protein production.

## Abbreviations

ACR: American College of Rheumatology
Akt: Protein kinase B
cAMP: Cyclic adenosine monophosphate
CCL18: Chemokine (C-C motif) ligand-18
cDNA: Complementary deoxyribonucleic acid
COL-1: Type I collagen
ECM: Extracellular matrix
Erk1/2: Extracellular signal-regulated kinases 1 and 2
ET-1: Endothelin-1
EULAR: European League Against Rheumatism
EUSTAR: European League Against Rheumatism scleroderma trials and research
FBS: Fetal bovine serum
FN: Fibronectin
GAPDH: Glyceraldehyde 3-phosphate dehydrogenase
HEPES: 4-(2-Hydroxyethyl)-1-piperazineethanesulfonic acid
HRP: Horseradish peroxidase
HS: Healthy subject
IL6: Interleukine-6
IP: Prostacyclin receptor
MAPK: Mitogen-activated protein kinase
NaCl2: Sodium chlorine
PAH: Pulmonary arterial hypertension
p-Akt: Phosphorylated protein kinase B
PBS: Phosphate buffer solution
p-Erk1/2: Phosphorylated extracellular signal-regulated kinases 1 and 2
qRT-PCR: Quantitative real-time polymerase chain reaction
S100A4: Fibroblast specific protein-1
SSc: Systemic sclerosis
SYBR: Syber
TGF: Transforming growth factor
α-SMA: Alpha smooth muscle actin
μM: Micromolar
